# Genetic variants associated with gastrointestinal symptoms in Fabry disease

**DOI:** 10.18632/oncotarget.13135

**Published:** 2016-11-05

**Authors:** Maria Teresa Di Martino, Francesca Scionti, Simona Sestito, Angela Nicoletti, Mariamena Arbitrio, Pietro Hiram Guzzi, Valentina Talarico, Federica Altomare, Maria Teresa Sanseviero, Giuseppe Agapito, Antonio Pisani, Eleonora Riccio, Osvaldo Borrelli, Daniela Concolino, Licia Pensabene

**Affiliations:** ^1^ Department of Experimental and Clinical Medicine, Magna Graecia University, Salvatore Venuta University Campus, Catanzaro, 88100 Italy; ^2^ Department of Medical and Surgical Sciences, Pediatric Unit, Magna Graecia University, Catanzaro, 88100 Italy; ^3^ ISN-CNR, Roccelletta di Borgia, Catanzaro, 88100 Italy; ^4^ Department of Medical and Surgical Sciences, Magna Graecia University, Catanzaro, 88100 Italy; ^5^ Departement of Nephrology University Federico II, Naples, 80138 Italy; ^6^ Department of Pediatric Gastroenterology, Great Ormond Street Hospital for Sick Children, University College of London (UCL), London, WC1E 6BT, UK

**Keywords:** Fabry disease, gastrointestinal symptoms, bile acids, DMET, genetic polymorphisms

## Abstract

Gastrointestinal symptoms (GIS) are often among the earliest presenting events in Fabry disease (FD), an X-linked lysosomal disorder caused by the deficiency of α-galactosidase A. Despite recent advances in clinical and molecular characterization of FD, the pathophysiology of the GIS is still poorly understood. To shed light either on differential clinical presentation or on intervariability of GIS in FD, we genotyped 1936 genetic markers across 231 genes that encode for drug-metabolizing enzymes and drug transport proteins in 49 FD patients, using the DMET Plus platform. All nine single nucleotide polymorphisms (SNPs) mapped within four genes showed statistically significant differences in genotype frequencies between FD patients who experienced GIS and patients without GIS: ABCB11 (odd ratio (OR) = 18.07, *P* = 0,0019; OR = 8.21, *P* = 0,0083; OR=8.21, *P* = 0,0083; OR = 8.21, *P* = 0,0083),SLCO1B1 (OR = 9.23, *P* = 0,0065; OR = 5.08, *P* = 0,0289; OR = 8.21, *P* = 0,0083), NR1I3 (OR = 5.40, *P* = 0,0191) and ABCC5 (OR = 14.44, *P* = 0,0060). This is the first study that investigates the relationships between genetic heterogeneity in drug absorption, distribution, metabolism and excretion (ADME) related genes and GIS in FD. Our findings provide a novel genetic variant framework which warrants further investigation for precision medicine in FD.

## INTRODUCTION

Fabry disease (FD [MIM:301500]), or Anderson-Fabry disease, is a rare X-linked lipid storage disorder caused by mutations in the *GLA* gene (MIM: 300644), encoding the lysosomal enzyme α-galactosidase A (α-Gal A). Deficient activity of α-Gal A leads to a progressive accumulation of neutral glycosphingolipids, predominantly globotriaosylceramide (Gb3), in the vascular endothelium of skin, kidney, nervous system, heart and other tissue with consequent multiorgan dysfunction [1]. In affected males, the clinical presentation of FD varies from a classical phenotype (null or minimal α-Gal A activity), including childhood-onset angiokeratoma, acroparesthesias, gastrointestinal disorders, hypohidrosisand characteristic corneal and lenticular opacities [2, 3] to adult-onset variants (residual α-GalA activity) with only cardiac [4] or renal [5] symptoms. Heterozygous females show a variable phenotype due to random X-inactivation [6]. However, published data showed a high degree of intra-familial phenotypic variability in patients carrying the same mutation [7]. Enzyme replacement therapy (ERT), with agalsidase alfa and agalsidase beta, has been reported to be well tolerated and effective in FD [8–11]. Gastrointestinal symptoms (GIS) are reported in approximately 60% of FD patients and have a profound negative effect on their quality of life. Post-prandial abdominal pain and diarrhea are the most prevalent manifestations, while less common GIS include nausea, vomiting, and early satiety [12]. Despite recent advances in clinical and molecular characterization of FD, the pathophysiology of the GIS is still poorly understood.

Here, we have evaluated the intervariability of GIS recurrence in a cohort of 49 FD patients. In order to determine the involvement of genetic factors unrelated to α-Gal A deficiency in the pathogenesis of GIS, we have performed a profiling of 1936 common and complex functional variants (SNPs, insertions, duplications, deletions) across 231 genes that encode for drug-metabolizing enzymes and drug transport proteins using the DMET Plus platform (Affymetrix, Inc., Santa Clara, CA, US).

## RESULTS

### Prevalence of gastrointestinal symptoms

The overall prevalence of GIS in the 49 evaluated patients was 24.5% (*n* = 12). Abdominal pain was present in 12 patients (24.5%) while diarrhea was reported in 11 patients (22.4%). One male patient complained only abdominal pain, all the other patients had more than one GI symptom: ten patients complained abdominal pain and diarrhea, one patient had heartburn, belching and bloating in addition to abdominal pain and diarrhea. The onset of GIS in our patients occurred at a median age of 9.1 years (range: 4–15 years). Ten out of the 12 patients with GIS were under ERT. After a period between 12 and 24 months of ERT, 40% patients (*n* = 4) reported an improvement in frequency and severity of GIS, while the remaining 60% (*n* = 6) reported no changes in GI manifestations after starting the treatment. Genotype-phenotype correlation revealed variability in the occurrence of GIS in related individuals carrying the same mutation.

### Correlation between genetic polymorphisms and gastrointestinal symptoms

All 49 patients passed QC metrics and produced useable genotypes. The average call rate was more than 95%. One SNP (rs2235033/*ABCB1*) failed to meet Hardy–Weinberg equilibrium (*p* < 0.05) and was excluded from further analysis. Of the remaining 1935 SNPs, 1025 SNPs were polymorphic in our population and were used in the association study. Nine SNPs in four genes unrelated to α-Gal A deficiency (*NR1I3*, *ABCC5*, *ABCB11*, *SLCO1B1*) resulted associated with GIS (Table 1).

**Table 1:** Genes and SNPs correlating with GIS in FD patients

Gene	SNP	Genotype	*P*-value
*NR1I3*	rs2501870	AG	0,0191
*ABCC5*	rs7636910	CT	0,0060
*ABCB11*	rs497692	CT	0,0083
	rs495714	CT	0,0019
	rs496550	CT	0,0083
	rs473351	CT	0,0083
*SLCO1B1*	rs2291075	CT	0,0065
	rs11045819	AC	0,0289
	rs2306283	AG	0,0083

**Figure 1:**
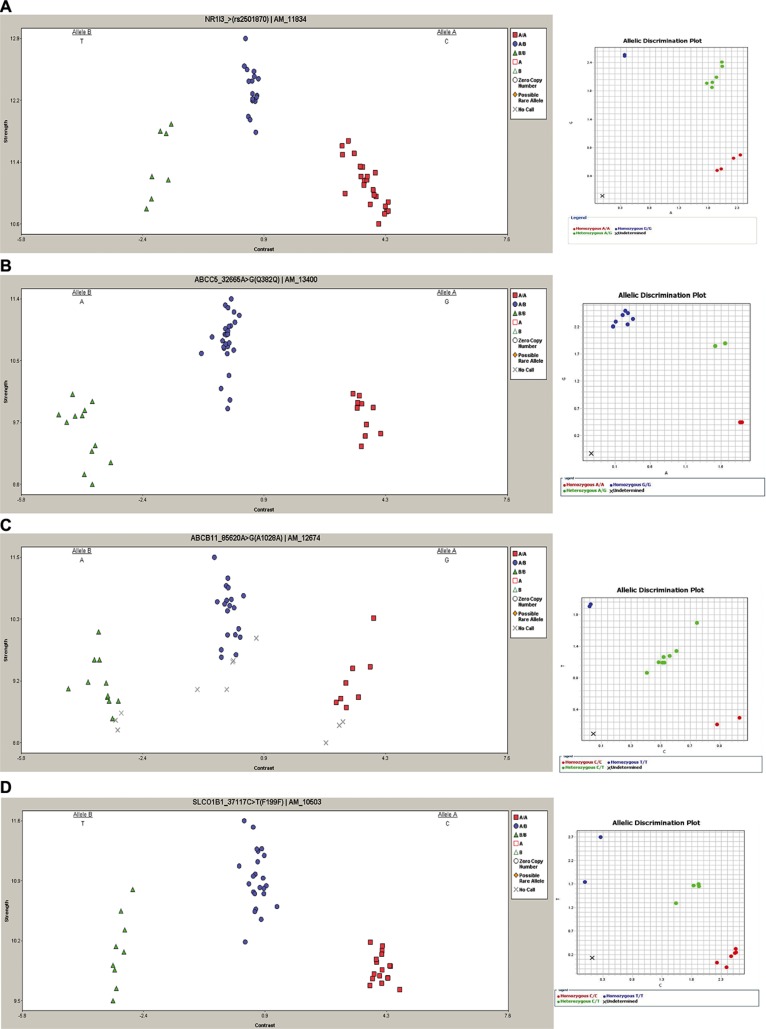
Representative DMET results and TaqMan SNP validation assay Left column: cluster graphs from DMET Console software displaying the signal data of all 49 samples genotyped for rs2501870, *NR1I3* (**A**), rs7636910, *ABCC5* (**B**), rs497692, *ABCB11* (**C**) and rs2291075, *SLCO1B1* (**D**). In rs2501870, rs497692 and rs7636910 alleles are reported in reverse orientation to genome. Right column: representative allelic discrimination plot (right column) generated from ViiA7 Real-Time PCR System software showing allelic symbols for rs2501870 (A), rs7636910 (B), rs497692 (C), rs2291075 (D) evaluation. In the plot the symbol “X” is referred to the No Template Control.

The heterozygous genotype AG (rs2501870) in the *NR1I3* gene and the heterozygous genotype CT (rs7636910) in the *ABCC5* gene resulted more frequent in patients with GIS compared to patients without GIS (67% *versus* 27% *P* = 0.0191, and 92% *versus* 43% *P* = 0.0060, respectively) (Table 2). Four *ABCB11* polymorphisms (rs497692, rs495714, rs496550, rs473351), in *linkage disequilibrium* (LD), significantly correlated with GIS, and the frequencies of the heterozygous genotypes CT were significantly higher in FD patients with GIS (CT = 84% *versus* 38% *P* = 0.0083, 92% *versus* 38% *P* = 0.0019, 84% *versus* 38% *P* = 0.0083 and 82% *versus* 38% *P* = 0.0083, respectively). In three *SLCO1B1* variants (rs2291075, rs11045819, rs2306283) the heterozygous genotypes (CT, AC and AG, respectively) showed statistically differences between the two groups, with higher frequencies in the FD patients with GIS (respectively 84% *versus* 35% *P* = 0.0065, 58% *versus* 22% *P* = 0.0289 and 84% *versus* 38% *P* = 0.0083, respectively), while the homozygous genotypes CC in rs2291075 and rs11045819 were associated with absence of GIS (respectively 43% *versus* 8% *P* = 0.0371 and 70% *versus* 34% *P* = 0.0392, respectively). These data were completely confirmed by TaqMan SNP Genotyping assays (Figure 1). All genotypes generated are reliable to 100% as we previously demonstrated [13].

**Table 2:** Polymorphisms associated with GIS in FD patients

				Genotype number (%)			
dsSNP ID*	Gene	Chr	Alleles	Patients with GIS (*N*=12)	Patients without GIS (*N* = 37)	*P*-value	OR (95% C.I.)
				GG = 4 (33.0)	GG =20 (54.0)		
rs2501870	*NR1I3*	1	A/G	AG = 8 (67.0)	AG = 10 (27.0)	0,0191	5.4 (1.33 to 21.95)
				AA = 0 (0.00)	AA = 7 (19.0)		
				TT = 1 (8.00)	TT=11 (30.0)		
rs7636910	*ABCC5*	3	C/T	CT =11 (92.0)	CT = 16 (43.0)	0,0060	14.44 (1.69 to 123.69)
				CC = 0 (0.00)	CC = 10 (27.0)		
				TT =1 (8.00)	TT = 13 (35.0)		
rs497692	*ABCB11*	2	C/T	CT = 10 (84.0)	CT = 14 (38.0)	0,0083	8.21 (1.57 to 43.08)
				CC = 1 (8.00)	CC = 10 (27.0)		
				TT = 1 (8.00)	TT = 10 (27.0)		
rs495714	*ABCB11*	2	C/T	CT = 11 (92.0)	CT = 14 (38.0)	0,0019	18.07 (2.10 to 155.49)
				CC = 0 (0.00)	CC = 13 (35.0)	0,0215	0.073 (0.004 to 1.32)
				TT = 1 (8.00)	TT = 12 (32.0)		
rs496550	*ABCB11*	2	C/T	CT =10 (84.0)	CT = 14 (38.0)	0,0083	8.21 (1.57 to 43.08)
				CC =1 (8.00)	CC = 11 (30.0)		
				TT = 1 (8.00)	TT = 11 (30.0)		
rs473351	*ABCB11*	2	C/T	CT =10 (82.0)	CT = 14 (38.0)	0,0083	8.21 (1.57 to 43.08)
				CC = 1 (8.00)	CC = 12 (32.0)		
				CC =1(8.00)	CC =16 (43.0)	0,0371	0.12 (0.01 to 1.02)
rs2291075	*SLCO1B1*	12	C/T	CT = 10 (84.0)	CT = 13 (35.0)	0,0065	9.23 (1.75 to 48.62)
				TT= 1 (8.00)	TT = 8 (22.0)		
				CC = 4 (34.0)	CC = 26 (70.0)	0,0392	0.21 (0.05 to 0.85)
rs11045819	*SLCO1B1*	12	A/C	AC = 7 (58.0)	AC = 8 (22.0)	0,0289	5.08 (1.26 to 20.36)
				AA = 1 (8.00)	AA = 3 (8.00)		
				AA = 1 (8.00)	AA = 13 (35.0)		
rs2306283	*SLCO1B1*	12	A/G	AG = 10 (84.0)	AG = 14 (38.0)	0,0083	8.21 (1.57 to 43.08)
				GG = 1 (8.00)	GG = 10 (27.0)		

## DISCUSSION

The pathophysiology of GIS in FD remains poorly understood although it is plausible that deposition of Gb3 in endothelial intestinal vasculature might cause enteric neuropathy. Moreover, Gb3 also accumulates in intestinal smooth muscle, and a direct myopathic effect, or combined myopathy and neuropathy, has been also suggested [14]. Enteropathy affecting the sympathetic and parasympathetic divisions of the autonomic nervous system is also possible, being autonomic nerve involvement previously reported in FD [15]. The peripheral neuropathy in FD manifests as neuropathic pain, reduced cold and warm sensation and possibly gastrointestinal disturbances [16].

In the present study, we investigated the relationships between genetic variants in ADME-related genes and GIS in FD, genotyping 49 FD patients with the DMET Plus microarray, that we have previously shown to be a powerful tool for the detection of genetic variants in ADME genes related to interindividual variability to drug response and/or toxicity [13, 17–20]. We identified nine SNPs within four genes (*NR1I3*, *ABCC5*, *ABCB11*, *SLCO1B1*) potentially associated with GIS.

We first found a positive correlation between the heterozygous genotype rs2501870 in the *NR1I3* gene and GIS. The *NR1I3* gene encodes the constitutive androstane receptor (CAR), which plays an essential and unique role in both controlling the mammalian sulfation pathways and facilitating bile acid (BA) detoxification and transport. The *NR1I3* rs2501870 is located in the 5′ region of the gene. Despite the absence of expression and/or function analysis, the presence of this SNP may result in the alteration of mRNA secondary structure, potentially influencing the transcription rate of *NR1I3*.

We also reported the association of the *ABCC5* rs7636910 with GIS. The *ABCC5* gene is a member of the ATP-Binding-Cassette family expressed primarily in the colon, liver, kidney and brain, and encodes the MRP5 protein implicated in the transport of cyclic nucleotides, including cAMP and cGMP [21]. The *ABCC5* rs7636910 does not change the glutamine at position 324 and so far its role on protein function and expression has not been elucidated. In addition we showed that four variants in *ABCB11* gene correlate with GIS. This gene encodes for the bile salt export pump (BSEP), responsible for hepatocyte-canaliculus export of conjugated and unconjugated BAs. Reduced function of BSEP, caused by inherited mutations or acquired factors, may lead to progressive intrahepatic cholestasis and severe liver disease [22]. BSEP expression is regulated by nuclear receptors. The aryl hydrocarbon receptor (AhR) and the pregnance X receptor (PXR) down-regulate BSEP expression while CAR and the farsenoid X receptor (FXR) up-regulate BSEP expression [23]. It also been shown that increased cAMP stimulates BSEP insertion into canalicular membrane [24]. Noteworthy, a previous study showed that ABCB11 overexpression in mice results in BA hypersecretion [25]. The *ABCB11* rs497692 results associated with altered transport function, promoting skipping of exon 24 and probably leading to dysfunction of the hepatobiliary bile salt export pump [26]. This SNP was previously associated with primary biliary cirrhosis, intrahepatic cholestasis of pregnancy and benign recurrent intrahepatic cholestasis [27–29]. The other *ABCB11* variants (rs495714, rs496550 and rs473351) are located in 3′ region of gene, including exon 28, but no clinical association has been previously reported for these SNPs.

Finally, we found three SNPs (rs2291075, rs2306283, rs11045819) in the *SLCO1B1* gene potentially associated with GIS. This gene encodes the organic anion transporting polypeptide1B1 (OATP1B1), a Na^+^-independent transporter expressed on the basolateral membrane of human hepatocytes that mediates the hepatic uptake of many endogenous compounds and xenobiotics, such as estrogen conjugates, BAs and statins. *In vitro* studies have shown that the rs2306283 and the rs2291075 are associated with reduced transport activity [30], while the rs11045819 shows unchanged transport function [31]. However, the strong LD between rs2306283 and rs2291075 leads to hypothesize a non-independent effect of the two SNPs. It has been reported that OATP1B1 expression is genotype-dependent and results higher in livers homozygous for rs2306283, rs11045819 and rs2291075. Moreover, livers that are heterozygous for rs11045819 have higher OATP1B1 expression than the wild-type, while the expression of OATP1B1 in livers carrying a single allele of rs2291075 and rs2306283 is not significantly different from the wild-type [32].

**Figure 2:**
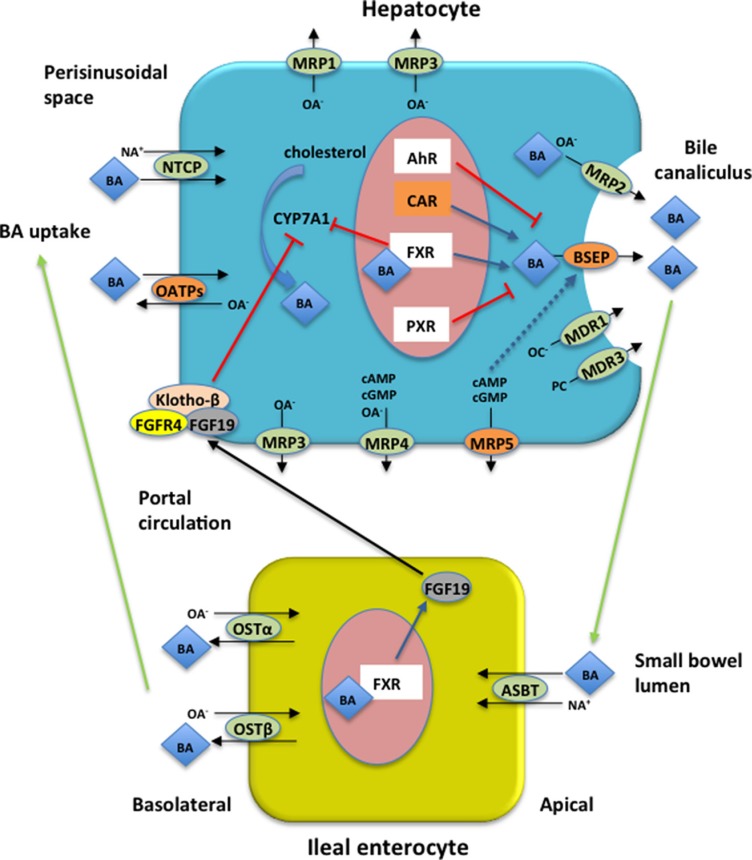
Enterohepatic circulation (EC) of bile acids (BAs) and proteins encoded by the four genes associated with GIS in FD patients Major BA transporters in human hepatocytes and enterocytes are shown. Blue arrows indicate up-regulation, red bars indicate down-regulation, while black arrows indicate transport across the cell. Proteins encoded by the four genes reported in the current study associated with GIS are represented in orange boxes. BAs are synthesized in hepatocytes from cholesterol by CYP7A1 which is thought to be the rate limiting step in BA synthesis. BAs active FXR to inhibit *CYP7A1* gene transcription. FXR induces intestinal hormone FGF19 which is released in the portal circulation and in the hepatocytes activates FGFR4/Klotho-β signaling inhibiting CYP7A1 activity. In hepatocytes BSEP excretes monovalent BAs in the bile canaliculus while divalent BAs and anionic conjugates are excreted via MRP2. MDR3 mediates secretion of phopholipids while organic cations are excreted via MDR1. Basolateral bile acid export system (MRP1, MRP3, MRP4, MRP5) excretes accumulated biliary constituents. In the terminal ilieum BAs are reabsorbed by ASBT and effluxed on the basolateral site via OSTα/β. BAs are taken up by the hepatocytes via NTCP and OATPs transport systems. BSEP expression is regulated by nuclear receptors (see text for details) and BSEP insertion into canalicular membrane is stimulated by cAMP (blue dashed arrow). AhR = aryl hydrocarbon receptor; ASBT = apical sodium bile salt transporter; BA = bile acid; BSEP = bile salt expert pump; CAR = constitutive androstane receptor; CYP7A1 = cholesterol 7a-hydroxylase; FGF19 = fibroblast growth factor 19; FGFR4 = FGF receptor 4; FXR = farsenoid X receptor; MRP1= multidrug resistance protein 1; MRP2 = multidrug resistance protein 2; MRP3= multidrug resistance protein 3; MRP4 = multidrug resistance protein 4; MRP5 = multidrug resistance protein 5; NA^+^ = sodium ion; NTCP = Na^+^-dependent taurocholate cotransport peptide; OATPs = Na^+^-independent organic anion transport proteins; OA- = organic anions; OC- = organic cations; OSTα/β = organic solute transporter α/β; PC = phosphatidylcholine; PXR = pregnance X receptor.

Patients with FD quite often report a previous diagnosis of diarrhea-predominant irritable bowel syndrome (IBS), a functional gastrointestinal disorder [33, 34]. In approximately 25% to 50% of the patients with IBS BAs induce diarrhea either through decreased ileal reabsorption or increased hepatic synthesis [35, 36]. The enterohepatic circulation (EC) of BAs is a physiological process mediated by a complex membrane transport system in the liver and intestine, regulated by nuclear receptors [37], as depicted in Figure 2. BAs are synthesized in the liver from cholesterol and are transported into bile ducts in conjugated form. They then accumulate and are stored in the gallbladder where they flow into the duodenum following meal-stimulated gallbladder contraction. After delivery into the intestinal lumen, the vast majority are reabsorbed by the distal ileum into the portal circulation and returned to the liver. A disruption of EC can alter the BA pool with consequent deregulation of the physiological process performed by BAs such as digestion and absorption of nutrient in the gut. The cause of BA diarrhea includes a deficiency in fibroblast growth factor 19 (FGF-19), a hormone produced in enterocytes in response to high intracellular concentration of BAs and that inhibits hepatic BA synthesis through the downregulation of the rate-limiting enzyme cholesterol 7a-hydroxylase (CYP7A1) [38]. However, recent pharmacogenomics evidence suggests that BA transporters are highly polymorphic leading to variable expression and function in humans [39, 40], so other potential causes of BA diarrhea can include genetic variations that affect the proteins involved in BA EC and synthesis. Theoretically, an excessive BA transport through the hepatocyte decreases their intracellular concentration and thus leading to enhanced BA biosynthesis; the increasing BA efflux that exceeds the normal capacity for ileal reabsorption can produce BA diarrhea.

In this study, we postulate that polymorphisms in *NR1I3*, *ABCC5*, *ABCB11* and *SLCO1B1* are associated with susceptibility to develop GIS in FD through an hypothetic mechanism that affect EC of BAs. However, further well-designed studies need to better investigate this hypothesis including methods for the diagnosis of BA diarrhea.

To our knowledge, this is the first study that uses a pharmacogenomics approach to explore genetic factors potentially involved in clinical manifestations in FD. Even if the effects associated to the identified variants remain to be investigated, our study is a hypothesis-generating early work. Further investigations will be required to better understand the role of these polymorphic variants in the pathogenesis of FD and a validation study in an independent FD patient group will be planned.

## MATERIALS AND METHODS

### Patients

49 patients (18 males; median age 40.3 years and 31 females; median age 39.4 years) from 18 families were enrolled in this study between December 2012 to April 2015 from the Department of Medical and Surgical Sciences, Pediatric Unit, *Magna Graecia* University of Catanzaro and from the Department of Nephrology, University *Federico II* of Naples. All patients were classically affected by FD and the diagnosis was confirmed by enzyme and/or molecular genetic analyses. Among the 49 FD patients, 12 experienced GIS while 37 did not. The mean age at diagnosis among FD patients with GIS was 31 years (range: 4–53), while among FD patients without GIS was 34,9 years (range: 2–60).

Moreover, 37 patients were under ERT at the time of enrolment in the study: 4 received infusions of agalsidase beta every 2 weeks at a dose of 1 mg/kg, while 33 received infusions of agalsidase alfa 0.2 mg/kg every other week, for an average of 5 years and 2 months (range: 11 months −11 years and 4 months). Baseline features of our FD patients and mutations are reported respectively in Table 3 and Table 4.

**Table 3:** Baseline details of FD patients

	Total	Male	Female
Total patients (no.)	49	18	31
Age (y; median SD)	40.3 ± 14.4	40.3 ± 10.1	39.4 ± 16.2
Under ERT	37	17	20
With Agalsidase alfa	33	16	17
With Agalsidase beta	4	0	4
Gastrointestinal symptoms (GIS)	12	7	6
abdominal pain	12	7	5
diarrhea	11	7	4
other GIS	1	1	0

**Table 4:** Mutations and phenotype of FD patients

Family	Mutations	phenotype
1	g.7192-7188delCAGCC(GGCTG)	classic
2	c.515G > A	classic
3	c.818T > C	classic
4	c.846_874delTC	classic
5	c.901C > G	classic
6	c.424T > C	classic
7	IVS2-76_80del5	classic
8	c.335G > A	classic
9	c.1184G > C	classic
10	IVS4+5G > T	classic
11	c.485G > A	classic
12	c.1066 C > T	classic
13	c.901C > T	classic
14	c.1066 C > T	classic
15	c.1133C > T	classic
16	c.67T >G	classic
17	c.680G > C	classic
18	c.1021dupG	classic

Laboratory and medical evaluations were undertaken at baseline to exclude other possible causes of GI complaints. All patients underwent clinical evaluation, laboratory tests such as full blood cell count, inflammatory markers (ESR and CRP), coeliac screening (antitransglutaminase and antiendomysial antibodies) including immunoglobulin levels, liver and kidney biochemical profiles, stool tests for infection (stool culture, examination for ova and parasites and for viral infection), fecal calprotectin, urinalysis and culture; moreover all patient underwent a trial with a lactose-free diet for 2 weeks or breath hydrogen testing for sugar malabsorption. No other cause of GIS was found in our FD patients. Frequency and consistency of GIS for each patient under ERT were evaluated every 2 weeks (at the time of ERT) by interviews and questionnaires as previously validated from our group [41]. Peripheral blood samples were collected from all participants after appropriate informed consent. The study was approved by the institutional review board in accordance with the Recommendation of the Declaration of Helsinki for biomedical research involving human subjects.

### DMET genotyping analysis

Genomic DNA was isolated from peripheral blood collected in EDTA tubes, using Perfect Pure DNA Blood kit (5 Prime) accordingly to the manufacturer’s recommendations and quantitated with Quanti-iT^TM^ PicoGreen^®^ dsDNA Assay kit (Invitrogen). All sample concentrations were normalized to 60 ng/μl and 1 μl of each DNA samples was analyzed using DMET plus arrays (Affymetrix Inc., Santa Clara, CA, US) according to the manufacturers’ indication as previously described by Di Martino *et al*. [17]. Briefly, the DMET Plus assay (Affymetrix Inc., Santa Clara, CA, US) uses Molecular Inversion Probe (MIP) technology to amplify the sequence-specific targets for each 1936 marker. The resulting target DNA were then enzymatic fragmented, labeled and hybridized to the arrays containing allele-specific oligonucleotides. Finally the DMET Plus arrays were scanned with the GeneChip Scanner 3000 (Affymetrix Inc., Santa Clara, CA, US) to obtain the intensity data by CEL files. Genotypes were calculated by DMET Console software version 1.1 using the Dynamic Genotype Boundaries algorithm (Affymetrix Inc., Santa Clara, CA, US). Patients with a call rate less than 95% were excluded from further analysis.

### TaqMan^®^ genotyping assay

DMET results were validated using pre-designed TaqMan^®^ Genotyping Assays (Thermo Fisher Scientific) provided in custom fast 96-well plates, according to manufacturer’s instructions. For each patient, we genotyped the following SNPs: rs2501870, rs7636910, rs2291075 and rs497692 (assay ID: C_16033318_20; C_25474215_30; C_1901690_1 and C_8813572_30, respectively). Reactions were performed in duplicate using an endpoint plate read on ViiA7 Real Time PCR System (Applied Biosystem). Thermal Cycling conditions included an initial step of denaturation at 95°C for 10 min followed by 40 cycles at 95°C for 15 sec and 60°C for 1 min. Allelic discrimination was made using the SDS software (Applied Biosystem) that plots Rn values (fluorescence of the reporter dye divided by the fluorescence of a passive reference dye) based on the fluorescence signals from each well.

### Statistical analysis

Genotypes for all SNPs were tested for deviation from Hardy-Weinberg equilibrium using a χ2 test and SNPs that were not in equilibrium (*P* < 0.05) were excluded. By DMET-Analyzer Tool software [42] were analyzed the genotype frequencies by two-tailed Fisher exact test in two groups: FD patients with GIS *versus* patients without GIS. Results of potential interest were limited to those in which the *p-value* was ≤ 0.05. Odds ratios (ORs) and corresponding 95% confidence intervals (CIs) were calculated for 2 × 2 table using Med Calc v12.3.0. No correction for multiple comparisons was performed.
